# The Impact of Entrepreneurial Optimism and Labor Law on Business Performance of New Ventures

**DOI:** 10.3389/fpsyg.2021.697002

**Published:** 2021-09-09

**Authors:** Shuai Li, Dongshuo Wu, Youxia Sun

**Affiliations:** ^1^Law School, East China Normal University, Shanghai, China; ^2^School of Social Development, East China Normal University, Shanghai, China

**Keywords:** entrepreneurial optimism, labor law, new ventures, business performance, impact mechanism

## Abstract

The purpose is to study the internal relationship between entrepreneurial optimism and business performance of new ventures, and the impact of entrepreneurial optimism on the business performance of new ventures. Based on the literature review, the hypotheses that entrepreneurial optimism has a positive impact on the business performance of new ventures and that labor law plays a mediating role in the impact are put forward. Then, the questionnaire is designed according to the maturity scale, and 200 questionnaires are collected. Finally, the descriptive statistical analysis, reliability analysis, exploratory factor analysis, confirmatory factor analysis, correlation analysis, and regression analysis of the theoretical model and hypothesis are carried out by using the statistical analysis software spsss22.0. The results show that each dimension of entrepreneurial optimism has a significant positive impact on the business performance of new ventures, and labor law plays a mediating role between them. This study provides a new idea for the establishment of the performance impact mechanism of new ventures and helps new entrepreneurs realize the importance of maintaining an optimistic attitude, improving the business performance of new ventures.

## Introduction

Entrepreneurship has great significance to the economic and social development of a country and region. It also plays an important role in promoting innovation and industrial restructuring, creating jobs, and shaping social culture (Ferreira et al., 2019). The government and the people worldwide pay close attention to the development of entrepreneurship and strive to create a good environment to promote mass entrepreneurship and innovation, stimulate the vitality of hundreds of millions of micro-market players, and develop new engines of economic development. In recent years, the continuous improvement of China’s market environment contributes to the increasing number of entrepreneurial activities, the all-round development of the entrepreneurial economy, and the popularity of entrepreneurship (Song and Jing, 2017). According to the report of Global Entrepreneurship Monitor, China’s entrepreneurial activities are continuously increasing, and it has become a country with active entrepreneurship. However, with the extensive development of entrepreneurial activities, the follow-up growth problems of enterprises follow, and it is related to the survival of new ventures. The immaturity of new enterprises and industry barriers poses a serious threat to them. Only a small number of new ventures develop into mature enterprises, and most of them die prematurely (Ghulam et al., 2017). Therefore, the business performance impact mechanism of new ventures is worthy of attention.

Wu and Wu et al. (2019) argued that the optimistic attitude of entrepreneurs is an important factor of new enterprises. Scholars in China and foreign counties made in-depth research on the impact mechanism of entrepreneurial attitude on the business performance of new ventures (Rankovi et al., 2020). However, there are few studies on entrepreneurs’ optimism, and a series of problems about how entrepreneurs’ optimism promotes and improves the business performance of new ventures are not studied and concerned (Qian et al., 2018). Dvouletý and Dvouletý believed that the business performance measurement indicators of new ventures should include sales rate, profit rate, and return on assets (Dvouletý, 2017; Diana and Maria, 2020). Sarraf and Nejad (2020) proposed BSC (Balanced Score Card) business performance evaluation system after the empirical research on several leading enterprises, which achieves a balance in many aspects, considers both financial indicators and non-financial indicators, and covers finance, customers, business management, and personnel training and development, breaking through the traditional method of measuring business performance only by financial performance [(Sarraf and Nejad, 2020). Messeghem et al. (2018) developed the business performance measurement scale based on BSC, which evaluates the business performance of new ventures from the aspects of financial performance, enterprise operation efficiency, customer and employee satisfaction, and loyalty. Most of the empirical results of enterprise performance evaluation show that it is not adequate to use only financial indicators to evaluate enterprise performance. Balanced score card no longer takes finance as the not only indicator to measure enterprise performance, but also considers non-financial indicators, achieving a balance in many aspects (Park et al., 2017; Dobrovič et al., 2018; Messeghem et al., 2018)]. The impact of policies on the enterprise economy is always a hot issue for scholars to study and discuss. Here, the labor law is introduced into the study of the relationship between entrepreneurial optimism and enterprise performance, and the impact of entrepreneurial optimism on the business performance of new ventures from a new perspective is discussed. Chen (2019) believed that the key to distinguishing charismatic leadership from non-charismatic leadership was whether they had foresight, self-confidence, and foresight, whether they could clearly state their goals, whether they could firm their faith, whether they could find another way, whether they understood change, and whether they were sensitive to the external environment (Chen, 2019). Yuan and Wu (2020) found that the unique qualities of successful entrepreneurs were sufficient knowledge to deal with market changes, risk-taking, innovation, scientific management skills, and cooperative spirit (Yuan and Wu., 2020; Liu and Chen, 2021).

The effect of the entrepreneur optimism on the business performance of new ventures in the new era of China is explored through the important intermediary of labor law, and the influence of entrepreneurship on the performance of enterprises from a new perspective and ideas is discussed to find a new path and solution for promoting China’s economic development. The main methods employed are literature analysis, questionnaire survey, and statistical analysis. It is very important to select the right and correct research methods and the correct statistical analysis methods for testing the theoretical model and hypothesis. Based on spss22.0 statistical analysis software, the research topic of the influence of entrepreneur optimism on the performance of new ventures is mentioned. The innovation points of the research are as: (1) to establish a performance evaluation model of new ventures from the perspective of entrepreneur optimism and labor law; (2) to formulate a series of concrete and feasible evaluation strategies according to the national conditions of domestic enterprises. The indicator system of this study is practical and referential and can provide research ideas and practical experience for the influencing factors of the performance of new ventures.

## Research Methods of Entrepreneurial Optimism and Labor Law on the Impact Mechanism of the Performance of New Ventures

### Research Method

It is very important to choose appropriate and correct research methods and data statistical analysis methods for testing the theoretical model and hypotheses. The empirical research method, literature analysis method (Melloncon and St. Amant, 2019), questionnaire survey method [(Rogoza et al., 2018), and statistical analysis method (Wu et al., 2019) are adopted. And SPSS22.0 statistical analysis software (Wu and Song, 2019) is also used to conduct descriptive statistical analysis, reliability and validity analysis (Wu et al., 2020), exploratory factor analysis (A, 2019)], confirmatory factor analysis, correlation analysis, and regression analysis.

### Data Processing

Based on the mature scale, the questionnaire design follows the principles of having a clear topic, reasonable questions and design, and the moderate number of questions, and easy to understand. The questionnaire covers several major provinces in China, including Guangxi, Guangdong, Zhejiang, Shandong, Anhui, Beijing, Sichuan, Heilongjiang, Guizhou, and Hainan. The research objects are the top managers of new ventures and some middle managers with decision-making ability. The data collection time is from September to December 2020. Because the measurement methods and tools of entrepreneurship optimism and business performance of new ventures are not perfect, it is difficult to quantify the evaluation of entrepreneurship optimism. According to the current situation and research purposes, most of the data are from entrepreneur training and some important meetings organized by entrepreneurs. There are 20 questions in the questionnaire, and 210 questionnaires are recovered, with an effective rate of 95.23%.

To test the relationship between entrepreneurial optimism and the business performance of new ventures, SPSS22.0 is used to conduct linear regression analysis: First, the demographic variables are taken as independent variables and the business performance of new ventures as dependent variables to construct regression model M1 (Wu et al., 2020). Second, the demographic variables are taken as control variables, entrepreneurial optimism as independent variables, and the business performance of new ventures as dependent variables to construct a regression model M2 (Wasowska, 2019).

### Research Hypothesis

#### Entrepreneurial Optimism and the Business Performance of New Ventures

Based on different research backgrounds, starting points, and research fields, scholars have a different understanding of the problem and entrepreneurs’ optimism. Wu and Wu. (2017) argued that entrepreneurs could update the old mode of production through “creative destruction, “innovate, restructure and recreate products, and production processes. Zheng et al. (2020) found that entrepreneur optimism referred to an individual characteristic of the entrepreneur himself, for example, entrepreneurs were cautious but decisive when facing risks. Zhou and Wu (2018) pointed out that entrepreneurship was a feature that could cope with uncertain changes and use changes as business opportunities. This study believes that “entrepreneurial optimism” refers to the personal characteristics of entrepreneurs, such as innovation, risk-taking, and initiative (Zhou and Wu, 2018). In the period of economic prosperity, entrepreneurs’ optimism spreads and their motivation are stimulated by profits. Entrepreneurs are willing to invest in different projects to pursue high profits. During the economic depression, pessimism arises and the entrepreneurs’ incentive force are determined by the expected value. In this case, entrepreneurs’ investment projects are becoming more and more the same, and herding behavior occurs. When it comes to the unilateral termination of the labor contract by the employer, there are almost no laws and regulations related to the performance appraisal of employees, except for the provisions of Article 39 and Article 40 of the labor contract law. On November 8, 2013, the judicial committee of the Supreme People’s court discussed and issued the no. 18 guiding cases. The key point is that the last rank in the employer’s grade assessment is not equal to the laborer’s “incompetence, “which does not meet the legal conditions for unilaterally terminating the labor contract, so the labor contract cannot be terminated unilaterally. Although this article alleviates the risk of being eliminated by employers, its deterrent effect is still weak, and it does not play a substantive role in the preferential protection of employees in practice. Chen et al. (2020) proposed that entrepreneurial optimism affects and improves the business performance of new ventures (Chen et al., 2020). Wang et al. (2020) believed that entrepreneurs’ optimism plays an important role in developing new products, looking for new business opportunities, and improving the core competitiveness of enterprises; entrepreneurs have the advantage of the market to obtain higher profits; and entrepreneurs’ language mode, attention, rational nature, and thinking style have a significant positive impact on the business performance of new ventures (Kim and Park, 2019; Li et al., 2021). Based on the above analysis, the following hypotheses are put forward:

*Hypothesis H1*: entrepreneurial optimism is positively correlated with the business performance of new ventures.

*Hypothesis H1a*: there is a positive correlation between entrepreneurs’ language mode and business performance of new ventures.

*Hypothesis H1b*: entrepreneurs’ rationality is positively related to the business performance of new ventures.

*Hypothesis H1c*: there is a positive correlation between entrepreneurs’ thinking style and the business performance of new ventures.

**Figure 1:**
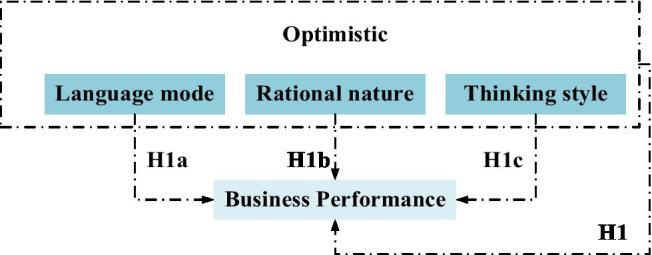
Relationship between entrepreneurial optimism and business performance of new ventures.

The relationship between entrepreneurial optimism and business performance of new ventures is shown in Figure 1.

To test the relationship between entrepreneurial optimism and the business performance of new ventures (Pfaller et al., 2020). SPSS22.0 is used to conduct linear regression analysis: First, demographic variables are taken as independent variables and the business performance of new ventures as dependent variables to construct regression model M1. Second, demographic variables are taken as control variables, entrepreneurial optimism as independent variables, and the business performance of new ventures as dependent variables to construct regression model M2. And SPSS 22.0 software is used to evaluate the reliability of the α coefficient, and the average value of the *α* coefficient is 0.871, which indicates that the questionnaire designed in this study has strong internal consistency and stability, and has high reliability. The questionnaire is reasonable and effective. Therefore, the next research can be carried out on this basis.

#### Entrepreneurs’ Optimism and Labor Law

Chang et al., (2020) and other foreign scholars do a lot of research in different periods from different perspectives. They study the relationship between enterprise leadership and the business performance of new ventures, and the relationship between entrepreneur’s characteristics, entrepreneur’s optimism, entrepreneur’s innovation behavior, and the labor law (Ban, 2019; Chen, 2019). Most empirical studies show that there is a positive correlation between different styles of leaders or entrepreneurs’ optimism and labor law (Chang et al., 2020). The optimistic attitude of entrepreneurs’ language mode, attention, rational nature, and thinking styles can play a positive role in influencing and guiding the improvement of labor law and also can play a promoting role in helping labor law play its functions better (Wang et al., 2020). Based on the above analysis, the following hypotheses are put forward:

*Hypothesis H2*: entrepreneurs’ optimistic attitude is positively related to the labor law.

*Hypothesis H2a*: language mode is positively related to labor law.

Hypothesis H2b: The nature of rationality is positively correlated with labor law.

Hypothesis H2c: The thinking style is positively related to labor law.

**Figure 2:**
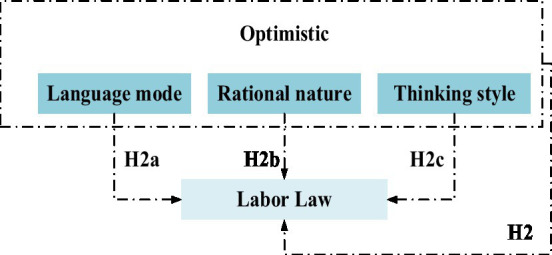
Relationship between entrepreneurs’ optimism and labor law.

The relationship between entrepreneurs’ optimism and labor law is shown in Figure 2.

#### Labor Law and Business Performance of New Ventures

Enterprises generally refer to legal persons or other social and economic organizations that use various production factors to provide goods or services to the market and carry out independent operation, self-management, self-reliance on profit and loss, and independent accounting. The types of enterprises are divided into three types: The first is the sole proprietorship enterprise, which is the enterprise that is invested, owned, and controlled by the individual, and the individual bears the operational risk and enjoys all the operating income. Second, partnership refers to the profit-making organization that each partner enters into a partnership agreement, jointly invests capital, and operates the business, shares the benefits, bears the risk, and several liabilities for the debts of the enterprise. Third, the company enterprise refers to the economic organization established, operated independently, responsible for profit and loss, and has legal personality established by investors with a legal person following the provisions of law. A company refers to a limited liability company and a joint-stock limited company established following the company law. From the definition of company law, the most direct classification of companies is limited liability companies and joint-stock limited companies. The joint-stock limited companies can be divided into listed companies and non-listed joint-stock limited companies (Deng et al., 2021).

**Figure 3:**
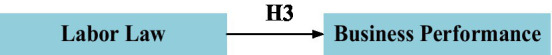
Relationship between labor law and the business performance of new ventures.

Labor law requires the establishment of a market economy system, changing the employment system of state-owned enterprises, breaking the restrictions of the market economy system, and promoting the marketization of labor relations. The market allocation principle and unified labor market rules of labor resources have been established, which opens the door for the free flow of labor force under market rules under the legal effect (Shpak et al., 2018); the basic labor standard conditions are taken as the legal bottom line, and all enterprises should implement them in a unified way; the labor relations should be established by signing labor contracts on an equal and voluntary basis, and the labor relations should be adjusted by collective negotiation; and all enterprises and workers should have equal market qualification, unified labor system rules, and labor relations regulation principles to establish a comprehensive social insurance system for all worker (Mihăilă et al., 2018). Based on the above theoretical analysis, the following hypothesis is put forward:

*H3*: There is a positive correlation between labor law and the business performance of new enterprises, as shown in Figure 3.

#### The Intermediary Role of Labor Law

Palme and Persson (2020) took the complaint rate, the proportion of employees punished, the number of contracts, negotiation time, absence rate, and labor attitude as the evaluation indicators of enterprise labor relations, and analyzed the relationship between enterprise labor relations and the performance of new ventures (Palme and Persson, 2020). The results show that the establishment of a high degree of trust in the working atmosphere and employee participation in the solution of operational problems helps to quickly reach an agreement on the issue of the collective tasks and reduce cumbersome work procedures. (Wu and Yenchun Jim, 2019; Campbell and Weststar, 2020) made a clear distinction between labor conflict mode and cooperative mode of labor relations and established measurement indicators to evaluate a large unionized manufacturing enterprise in the United States. Taking conflict frequency, conflict resolution, formal and informal complaint, work autonomy, and work feedback as evaluation indicators, the impact of labor relations on enterprise cost and productivity are analyzed (Campbell and Weststar, 2020). Common interests make it easy to form an atmosphere with clear objectives, active employees, strong participation, and overall unity and cooperation within the enterprise, which plays a role in easing the conflict between the labor force and capital, broadening the space for labor and capital cooperation (Lu and Xie, 2018). The basis of establishing cooperative labor relations is that employees, trade union organizations representing employees, employers, and their organizations can negotiate on an equal basis, conduct collective bargaining, and trust and respect each other. Also, enterprises provide employees with good employment security and working conditions, participation in management systems and measures, and smooth channels for employees to speak and appeal, and long-term interests, which is conducive to the formation of cooperative labor relations and has a positive impact on the performance of new ventures (Hazzi and Hammami, 2019; Chen et al., 2020). Based on the above theoretical analysis, the following hypotheses are put forward:

*Hypothesis H4*: entrepreneurial optimism has a positive impact on the performance of new ventures through the mediating role of labor law.

*Hypothesis H4a*: language mode has a positive effect on the performance of new ventures through the mediation of labor law.

*Hypothesis H4b*: rational nature has a positive effect on the performance of new ventures through the mediating role of labor law.

*Hypothesis H4c*: thinking style has a positive effect on new venture performance through the mediation of labor law.

**Figure 4:**
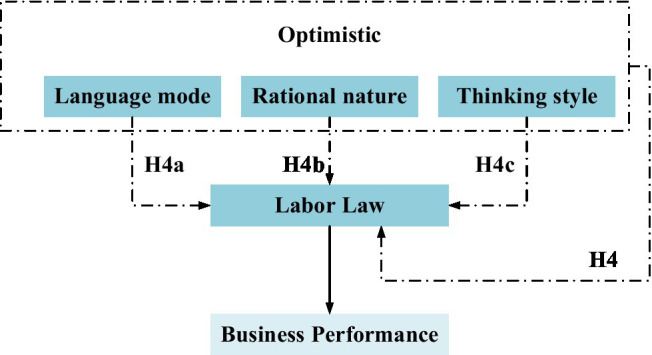
The mediating role of labor law.

The mediating role of labor law is shown in Figure 4.

## Research Results and Analysis

### The Impact of Corporate Optimism on the Performance of New Ventures

**Figure 5:**
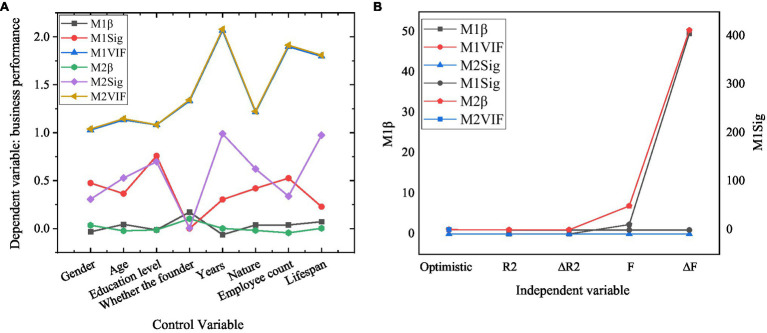
Test of the relationship between entrepreneurial optimism and business performance of new ventures.

The relationship between entrepreneurial optimism and business performance of new ventures is shown in Figure 5.

Figure 5 shows that after the influence of demographic variables is controlled, the entrepreneur optimism significantly affects the performance of the new ventures. *β* is the regression coefficient, Sig is the significance, and VIF is the variance inflation factor. Specifically, the *F* value of the model reaches significance at the level of *p*<0.05, which indicates that the fitting of the regression model is better; all the values of VIF are less than five, showing that there are no multiple collinearities between demographic variables and entrepreneur optimism as independent variables. *R*^2^ is 0.473, and entrepreneur optimism could be explained to be 47.3%. The regression coefficient of entrepreneur optimism (*p*<0.001) is significant, which shows that there is a significant positive correlation between entrepreneur optimism and the performance of the new ventures. Therefore, hypothesis H1 is verified.

The results show that the three dimensions of entrepreneurial optimism: language mode, rational nature, and thinking style significantly affect the business performance of new ventures. *F* values are significant when *p*<0.05, which indicates that the regression model has good fitting; VIF values are less than five, which indicates that there is no multicollinearity among the three dimensions of demographic variables and entrepreneurial optimism. *R*^2^ is 0.474, and the explanatory variables of entrepreneurial optimism are 47.4%. The *p* values of language mode, rational nature, and thinking style are all less than 0.001, and the regression coefficient is significant. This shows that there is a significant positive correlation between the three dimensions of entrepreneurial optimism and the business performance of new ventures. In this case, H1a, H1b, and H1c are verified.

### The Influence of Entrepreneurs’ Optimism on Labor Law

**Figure 6:**
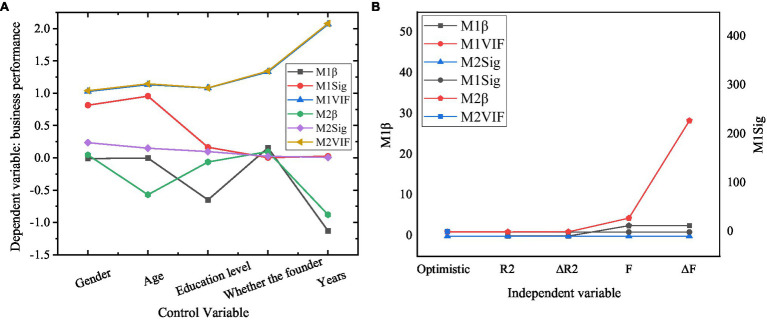
Relationship between entrepreneurs’ optimism and labor law.

The relationship between entrepreneurs’ optimism and labor law is shown in Figure 6.

Figure 6 shows that entrepreneurs’ optimism has a significant impact on labor law. *F* value is significant when *p*<0.01, which indicates that the regression model has a good fitting; VIF is less than five, indicating that there is no multicollinearity between demographic variables and entrepreneurial optimism as independent variables in the regression model. *R*^2^ is 0.342, entrepreneurial optimism can be explained by 34.2%, and the regression coefficient of entrepreneurial optimism (*p*<0.001) is significant. This indicates that there is a significant positive correlation between entrepreneurial optimism and labor law, and hypothesis H2 is verified.

The results show that the *p* value of language mode, rational nature, and thinking style is less than 0.001, and the regression coefficient is significant. This shows that there is a significant positive correlation between the three dimensions of entrepreneur optimism, namely, language mode, rational nature, and thinking style, and labor law. Therefore, H2a, H2b, and H2c are verified.

### Impact of Labor Law on Business Performance of New Ventures

According to the above methods, the relationship between labor law and the business performance of new ventures is tested, and the test results are shown in Table 1.

**Table 1:** Relationship between labor law and business performance of new ventures.

	Dependent variable: business performance					
	Model M1			Model M2		
	*β*	Sig	VIF	*β*	Sig	VIF
**Control Variable**						
Gender	−0.033	0.475	1.028	0.024	0.444	1.028
Age	−0.044	0.365	1.134	0.045	0.188	1.134
Education level	−0.15	0.758	1.082	0.031	0.364	1.087
Whether the founder	0.173^**^	0.002	1.331	0.066	0.078	1.355
Years	−0.066	0.303	2.067	0.034	0.478	2.079
Nature	0.038	0.419	1.215	0.015	0.686	1.216
Employee count	0.038	0.526	1.897	0.012	0.788	1.888
Lifespan	0.072	0.228	1.796	0.045	0.301	1.795
**Independent variable**						
Optimistic				0.693^***^	0.000	1.042
*R* ^2^	0.038^**^	0.018		0.478^***^	0.000	
∆*R*^2^	0.22^*^	0.018		0.461^***^	0.000	
*F*	2.365^*^	0.018b		54.575^***^	0.000c	
∆*F*	2.365^*^	0.018		455.002^***^	0.000	

* *p*<0.05;

** *p*<0.01;

*** *p*<0.001.

Table 1 shows that labor law has a significant impact on the business performance of new ventures. *F* values are significant when *p*<0.05, which indicates that the regression model has good fitting. VIF is less than five, indicating that there is no multicollinearity between demographic variables and labor law included in the regression model. *R*^2^ is 0.478, labor law can be explained variation is 47.8%, and the regression coefficient of entrepreneurial optimism (*p*<0.001) is significant, which indicates that there is a significant positive correlation between labor law and the business performance of new ventures. H3 is verified.

### Test of the Intermediary Role of Labor Law

**Figure 7:**
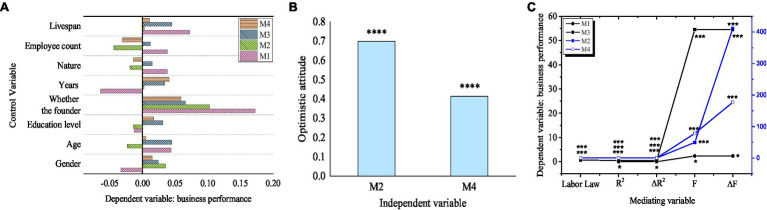
Test of the mediating effect of labor law (**A**, **B**, and **C** are control variable, independent variable, and mediating variable coefficient, respectively).

The mediating role of labor law between entrepreneurial optimism and business performance of new ventures is shown in Figure 7.

Figure 7 shows that the coefficient of entrepreneur optimism of the independent variable decreases significantly after the intermediary variable is added, which proves that there is a partial intermediary relationship between entrepreneur optimism and the performance of new ventures in labor law. Therefore, hypothesis H4 is verified. A further test should be conducted to reveal whether the labor law plays a mediating role in the three dimensions of entrepreneurial optimism, namely, language mode, rational nature, and thinking style, as well as the performance of new ventures.

**Figure 8:**
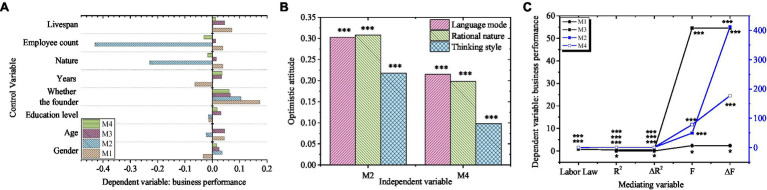
Test of the mediating effect of labor law (**A**, **B**, and **C** are control variable, independent variable, and mediating variable coefficient, respectively).

Figure 8 shows that the coefficients of the three dimensions (language mode, rational nature, and thinking style) of entrepreneurial optimism as the independent variables decrease significantly after the mediating variables are added, which proves that there is a partial mediating relationship between the three dimensions of entrepreneurial optimism and the performance of new ventures. Therefore, H4a, H4b, and H4c are verified.

### Summary and Discussion of Hypothesis Test Results

There are four hypotheses and nine sub-hypotheses, with a total of 13 hypotheses, all of which pass the tests, and the test conclusions are summarized in Table 2.

**Table 2:** Summary of test results of research hypotheses.

Serial number	Inspection content	Hypothetical result
H1	Entrepreneurs’ optimism is positively correlated with corporate performance	Supported
H1a	Language mode is positively related to corporate performance	Supported
H1b	The nature of rationality is positively correlated with corporate performance	Supported
H1c	Thinking style is positively related to corporate performance	Supported
H2	Entrepreneurs’ optimism is positively correlated with labor law	Supported
H2a	The language model is positively related to labor law	Supported
H2b	The nature of rationality is positively correlated with labor law	Supported
H2c	The tinking mode is positively related to labor law	Supported
H3	There is a positive correlation between labor law and the business performance of new enterprises	Supported
H4	Entrepreneurs’ optimism positively affects corporate performance through the intermediary role of labor law	Supported
H4a	Language mode positively affects corporate performance through the intermediary role of labor law	Supported
H4b	The nature of rationality positively affects corporate performance through the intermediary role of labor law	Supported
H4c	Thinking style positively affects corporate performance through the mediating role of labor law	Supported

The empirical results show that all 13 hypotheses pass the tests. The results are discussed as follows:

First, hypotheses H1, H1a, H1b, and H1c are tested. The entrepreneurial performance is influenced by the entrepreneur’s optimistic attitudes. The more significant the role of language mode, rational nature, and thinking style of entrepreneurs is, the better the performance of new ventures is. These three dimensions have different effects on the business performance of new ventures. Language mode and rational nature have more positive effects on the business performance than thinking styles (Sulakhe and Bakre, 2019).

Second, H2, H2a, H2b, and H2c are verified. Entrepreneurs’ optimism and its three dimensions are positively correlated with labor law. The language mode, rational nature, and thinking style of entrepreneurs play an increasingly important role in labor law, among which the thinking style plays an increasingly important role (Lin and Pursiainen, 2021).

Third, H3 is verified. There is a significant positive correlation between labor law and the business performance of new ventures. The stronger the labor law is, the better the performance of new ventures is.

Fourth, H4, H4a, H4b, and H4c are verified. Labor law plays an intermediary role between entrepreneurial optimism and the business performance of new ventures. Entrepreneurial optimism also has a significant positive impact on the business performance of new ventures. After the labor law variables are added, the regression coefficient becomes larger and the influence is enhanced. Therefore, labor law plays a strong mediating role between the three dimensions of entrepreneurs and the business performance of new ventures (Liang, 2019).

Through empirical analysis, it is found that entrepreneurial optimism plays a positive role in promoting enterprise performance, and entrepreneurial optimism positively promotes enterprise performance through employee innovation behavior. The cultivation of entrepreneurs’ optimism should be paid more attention; then, their innovation behavior of ordinary employees is stimulated, and the transmission and influence between the two are explored, maximizing the effectiveness of the two, and jointly promoting the improvement of enterprise performance, the transformation and upgrading of Chinese enterprises, and China’s economic development (Liu et al., 2021).

## Conclusion

Based on the labor law, the relationship between entrepreneurial optimism and the business performance of new ventures is discussed, and relevant hypotheses are made. And specific examples are used to verify the relationship between entrepreneurial optimism and business performance, as well as the role of labor law between them. Entrepreneurial optimism plays a positive role in promoting the business performance of new ventures, and it positively promotes the performance of new ventures through employees’ innovative behavior (Chen, 2018; Shen et al., 2019). Labor law plays an intermediary role in the relationship between entrepreneurs’ optimism and the business performance of new ventures, which requires that the cultivation of entrepreneurial optimism should be emphasized, the innovation behavior of employees should be encouraged, and influence between the entrepreneur’ optimism and the employee’s innovation should be paid attention to as well, maximizing the effectiveness of the two and jointly promoting the business performance of new ventures. This plays an important role in promoting the healthy development of enterprises in China. However, there are still some shortcomings of the research: (1) Although the study proves that entrepreneurial optimism has a positive impact on the performance of enterprises, the negative impact of entrepreneurs’ pessimism on the performance of new ventures is not discussed; (2) The existing model does not involve financial indicators, which may be one of the aspects affecting the performance of new ventures. In the future, in-depth analysis and research from these two perspectives should be conducted to provide a theoretical basis for the sustainable development of enterprises.

## Data Availability Statement

The raw data supporting the conclusions of this article will be made available by the authors, without undue reservation.

## Ethics Statement

The studies involving human participants were reviewed and approved by East China Normal University Ethics Committee. The patients/participants provided their written informed consent to participate in this study.

## Author Contributions

All authors listed have made a substantial, direct and intellectual contribution to the work, and approved it for publication.

## Conflict of Interest

The authors declare that the research was conducted in the absence of any commercial or financial relationships that could be construed as a potential conflict of interest.

## Publisher’s Note

All claims expressed in this article are solely those of the authors and do not necessarily represent those of their affiliated organizations, or those of the publisher, the editors and the reviewers. Any product that may be evaluated in this article, or claim that may be made by its manufacturer, is not guaranteed or endorsed by the publisher.

## Appendix

**Table:** The specific contents of the questionnaire are as follows:

S. No.	The impact of entrepreneurial optimism on the business performance of new ventures
	Dear ladies and gentlmen, Thank you very much for your participation in the survey. The purpose of this questionnaire is to study the formation path of the busines performance of new ventures and mainly explore the impact of entrepreneurs’ optimism on the business performance of new ventures. Your opinions and answers are very important to us. We promise that your answers will be used for academic research only and will never be abused. You can choose the corresponding answer according to your heart. Thank you again for your participation!
1.	Gender: Male () Female ()
2.	Age: Under 30 () 31~45() Above 45 ()
3.	Occupation
4.	Tpye of your enterprise: State-owned enterprises () Private enterprises () Joint venture or wholly foreign owned () Others ()
5.	Number of the employees of your enterprise: Less than 100 () 101~1,000 () 1,001–3,000 () More than 3,000 ()
6.	Years of the establishment of your enterprise: Less than 1year () 1–5years () 5–10years () More than 10years ()
7.	Developmental period of your enterprise: (Initial period) All kinds of resources are still scarce, and survival is the preferred goal () (Growth period) the scale of organization and production develops and begins to enter a period of rapid development () (Mature period) the profit are relatively stable, and they begin to expand scale expansion and set more grand social goals () (Recession) the company has passed the peak period of development, showing signs of recession and facing transformation ()
8.	Your department is as: R and D () Marketing () Executive () Financial accounting () Production ()
9.	Environmental uncertainty: There are uncertainties and challenges in the work () Enterprises can actively introduce new technologies to cope with environmental uncertainty ()
10.	Attitudes of the entrepreneurs toward innovative spirit (multiple choice): Fund is often used to develop new projects () The importance is attached to the development of new and innovative products () Technological innovation based on research results is quickly accepted () Enterprise strategy emphasizes the capability R and D, technology leadership, and innovation of product or service () New ideas of all levles are Encouraged () The enterprises have entered new markets ()
11.	Risk-taking spirit of the entrepreneurs (multiple choice) Willing to invest in new business opportunities () Prefer high risk and high return projects () Tend to take bold and rapid actions to achieve goals () Have a positive attitude to increase the possibility of obtaining potential opportunities ()
12.	Entrepreneurs’ optimism (multiple choices): Have Good language mode () Have positive rational nature () Possess active thinking style ()
13.	The positive impact of the improvement of labor law on entrepreneurs’ management Great () Average () little () no ()
14.	Competitiveness of entrepreneurs Take the lead and not just react to our opponent’s actions () Play a leading role in the innovation of the industry () Always show a competitive attitude, trying to beat competitors ()
